# Visceral variant of Lemierre's syndrome presenting with liver abscess, hepatic vein thrombophlebitis, and septic pulmonary emboli

**DOI:** 10.1093/omcr/omag160

**Published:** 2026-08-22

**Authors:** Huei-Chu Cheng, Ying-Lin Tan, Li-Wei Lin, Chee-Fah Chong

**Affiliations:** Emergency Department, Shin-Kong Wu Ho-Su Memorial Hospital, No. 95, Wenchang Road, Shilin District, Taipei City 111045, Taiwan; School of Medicine, Fu Jen Catholic University, No. 510, Zhongzheng Road, Xinzhuang District, New Taipei City 242062, Taiwan; Emergency Department, Shin-Kong Wu Ho-Su Memorial Hospital, No. 95, Wenchang Road, Shilin District, Taipei City 111045, Taiwan; Emergency Department, Shin-Kong Wu Ho-Su Memorial Hospital, No. 95, Wenchang Road, Shilin District, Taipei City 111045, Taiwan; School of Medicine, Fu Jen Catholic University, No. 510, Zhongzheng Road, Xinzhuang District, New Taipei City 242062, Taiwan; Emergency Department, Shin-Kong Wu Ho-Su Memorial Hospital, No. 95, Wenchang Road, Shilin District, Taipei City 111045, Taiwan; School of Medicine, Fu Jen Catholic University, No. 510, Zhongzheng Road, Xinzhuang District, New Taipei City 242062, Taiwan

**Keywords:** Lemierre's syndrome, *fusobacterium necrophorum*, hepatic vein thrombophlebitis, pyogenic liver abscess, septic pulmonary emboli

## Introduction

Lemierre's syndrome, first described by André Lemierre in 1936, is classically defined by oropharyngeal sepsis, internal jugular vein thrombophlebitis, and hematogenous dissemination of anaerobic organisms, predominantly *Fusobacterium necrophorum*, to distant organs. The lungs are the most frequent site of metastatic seeding, with pulmonary infarctions and cavitating nodules representing characteristic findings [1, 2]. Despite its severity, the syndrome remains underrecognized due to its rarity, and the prototypical presentation in young adults can be mistaken for uncomplicated pharyngitis before systemic deterioration occurs [2, 3].

Conceptually, however, the syndrome is better understood as a pathophysiologic triad rather than an anatomic one: a primary focus of anaerobic infection, contiguous septic thrombophlebitis of the draining vein, and downstream metastatic septic embolization from that infected thrombus. It is this sequence, rather than the oropharyngeal site of origin per se, that defines the entity. The visceral (abdominal) variant of Lemierre's syndrome, in which the primary infectious focus resides within the abdomen or pelvis rather than the oropharynx, has been reported only in isolated case series and small case reports [4, 5]. In these atypical cases, the portal, mesenteric, or hepatic venous system assumes the role of the internal jugular vein as the conduit for septic thrombophlebitis and downstream embolic seeding [5]. Because the oropharyngeal hallmark is absent by definition in this variant, diagnosis is frequently delayed and anaerobic bacteremia underappreciated. We report this case to expand clinician awareness of the visceral variant of Lemierre's syndrome and to highlight key diagnostic and management considerations.

## Case report

A 32-year-old previously healthy male presented with a 5-day history of progressive fatigue, myalgia, anorexia, headache, and intermittent high fevers, followed by nausea and a retrosternal burning sensation. He denied upper respiratory symptoms, sore throat, cough, diarrhea, or abdominal pain. He specifically denied anorectal pain, tenesmus, rectal discharge, or rectal bleeding, and digital rectal examination and perianal inspection were unremarkable. He had no relevant medical history and no regular medications. He is a man who has sex with men (MSM), recorded as a potentially relevant epidemiologic factor; he had no recent travel, animal exposures, or wilderness activities. HIV, hepatitis A, B, and C serologies, and syphilis testing (RPR) were negative or nonreactive. Atypical pathogen serologies obtained during the fever work-up (*Mycoplasma pneumoniae* IgM, cytomegalovirus IgM, and Epstein–Barr virus viral capsid antigen IgM) were also negative; *Chlamydia pneumoniae* IgG was mildly elevated (13.83; reference < 9.0) with a negative IgM, a pattern consistent with prior rather than acute infection.

On arrival, he was critically ill: temperature 39.5°C, blood pressure 81/44 mmHg, heart rate 113 bpm. Laboratory findings revealed an elevated C-reactive protein (21.25 mg/dL [212.5 mg/L]), elevated lactate (23.5 mg/dL [2.6 mmol/L]), leukocytosis (11.2 × 10^3^/μL [11.2 × 10^9^/L]) with 91% neutrophils, and severe thrombocytopenia (platelets 14 × 10^3^/μL [14 × 10^9^/L]). Coagulation studies showed markedly elevated D-dimer (4825 ng/mL FEU [4.83 mg/L FEU]), elevated fibrinogen (682 mg/dL [6.82 g/L]), and a positive lupus anticoagulant (dilute Russell's viper venom time [dRVVT] normalized ratio 1.29; reference < 1.21). Hepatic dysfunction was evidenced by hyperbilirubinemia (total bilirubin 6.44 mg/dL [110.1 μmol/L]; direct 4.98 mg/dL [85.2 μmol/L]), with mildly elevated aminotransferases (AST 48 U/L; ALT 75 U/L) and a normal alkaline phosphatase (109 U/L). Additional findings included hyperglycemia (glucose 124 mg/dL [6.9 mmol/L]), mildly elevated creatinine (1.19 mg/dL [105.2 μmol/L]) with a blood urea nitrogen of 10 mg/dL [3.6 mmol/L urea], hyponatremia (sodium 128 mmol/L), and hypokalemia (potassium 3.1 mmol/L).

**Figure 1:**
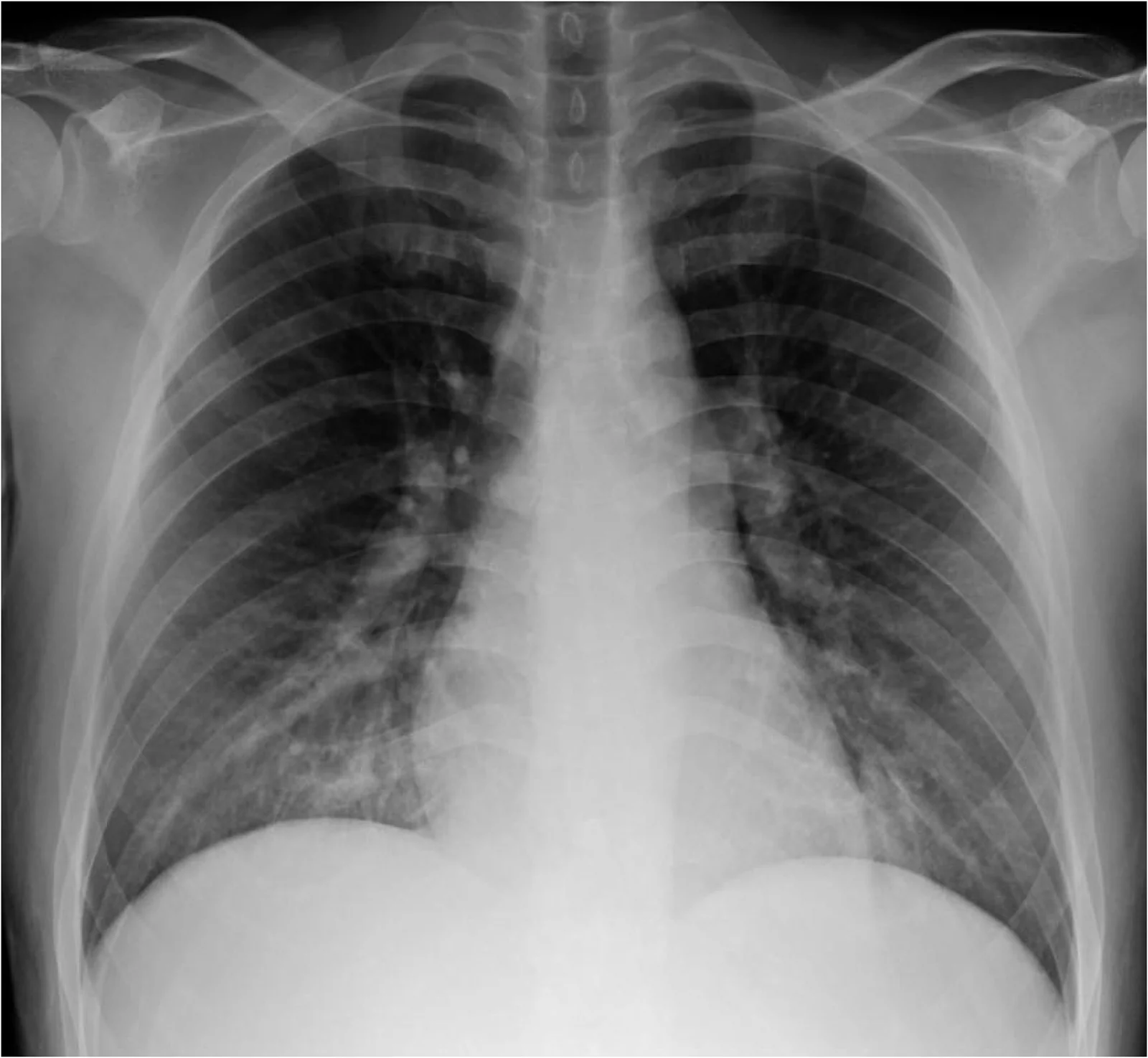
Chest radiograph demonstrating bilateral basal infiltrates and opacities corresponding to the septic pulmonary emboli demonstrated on chest CT (Fig. 2A).

**Figure 2:**
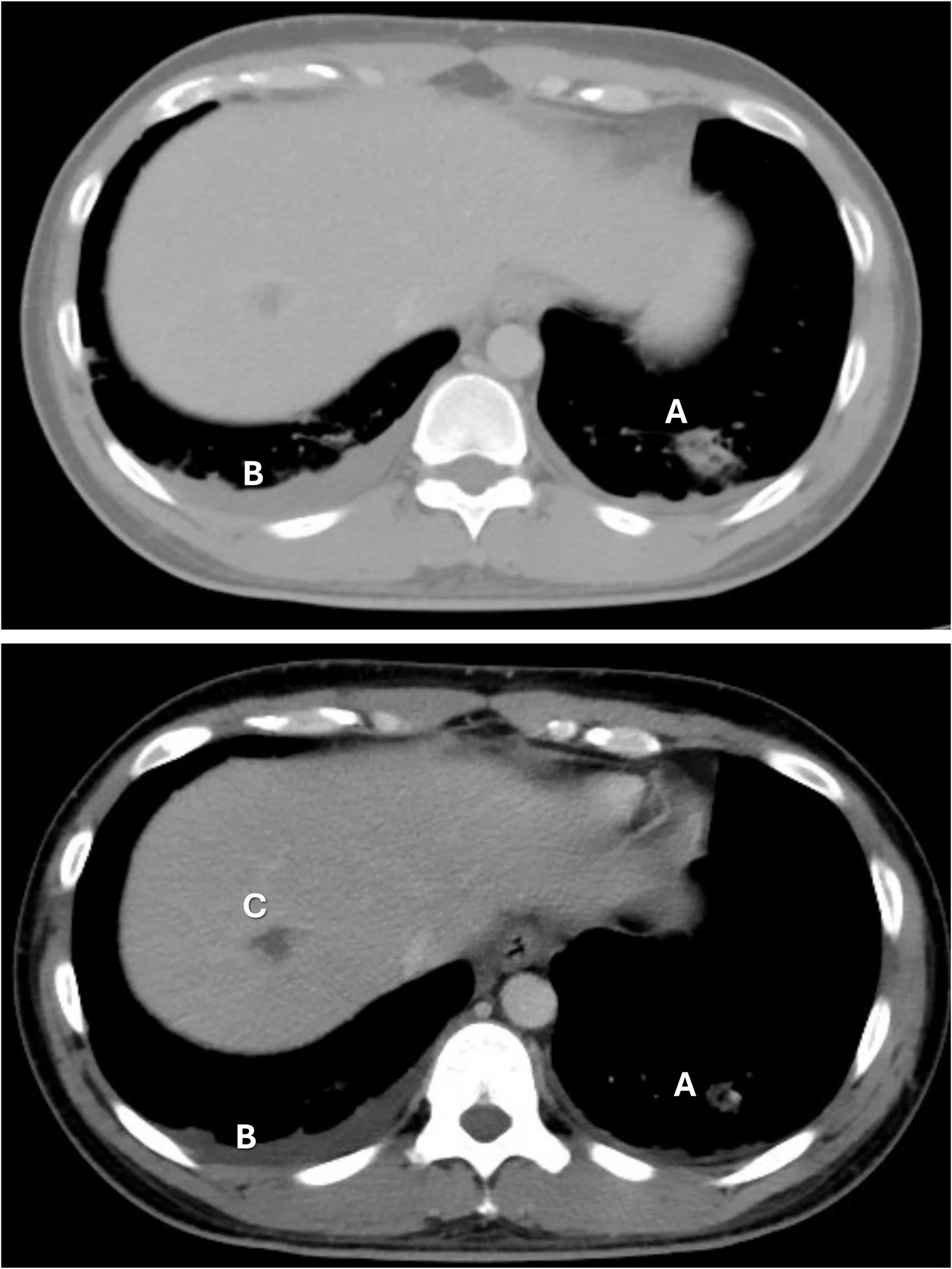
Axial chest and abdominal CT: (A) multiple bilateral peripheral wedge-shaped pulmonary nodules, a pattern characteristic of septic emboli; (B) small right pleural effusion; (C) ring-enhancing hypodense lesion in hepatic segment 8 (S8) dome, consistent with a pyogenic abscess.

**Figure 3:**
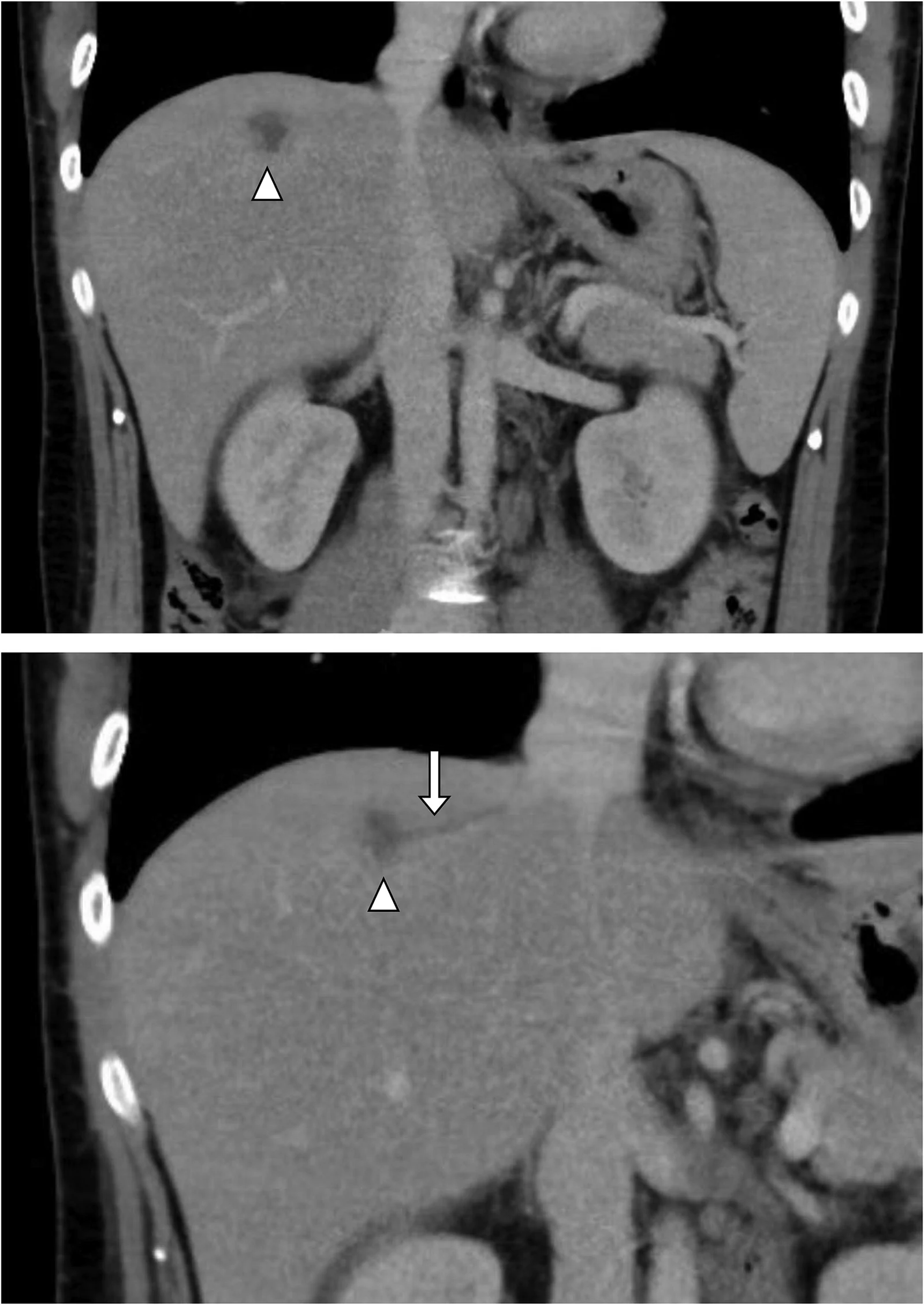
Coronal abdominal CT demonstrating the hepatic S8 dome abscess (arrowhead) with adjacent hepatic vein thrombophlebitis (arrow), identified by wall thickening and enhancement of the hepatic vein with internal low density, findings supporting infected (septic) thrombus rather than bland thrombosis, which would instead show an extensive thrombus burden, venous obstruction, or a hepatic perfusion abnormality, none of which were present on this study.

Chest radiograph demonstrated bilateral basal infiltrates and opacities (Fig. 1). Contrast-enhanced CT of the chest, abdomen, and pelvis identified a 1.5-cm hypodense hepatic dome collection consistent with pyogenic liver abscess with regional hepatic vein thrombophlebitis and bilateral multifocal peripheral wedge-shaped pulmonary nodules consistent with septic emboli (Fig. 2). The hepatic vein finding was characterized by wall thickening and enhancement with internal low density consistent with infected thrombus, without imaging features of bland thrombosis such as an extensive thrombus burden, venous obstruction, or a hepatic perfusion abnormality (Fig. 3). Importantly, the same study demonstrated a patent portal and superior mesenteric venous system without intraluminal thrombus, periportal inflammatory change, or gas, and no rectal, sigmoid, or colonic wall thickening, pericolic fat stranding, perirectal fluid, or intra-abdominal or pelvic collection. Echocardiography revealed right ventricular volume overload, mild pulmonary hypertension, and an engorged inferior vena cava without valvular vegetations. Otolaryngologic evaluation, including bedside pharyngolaryngoscopy, excluded an oropharyngeal or deep neck source, and no *F. necrophorum* was identified from a pharyngeal source, effectively excluding classic Lemierre's syndrome; dedicated imaging of the internal jugular veins was not performed, and the venous findings in this case were characterized on abdominal CT alone.

Empiric intravenous meropenem and doxycycline were initiated on admission for broad-spectrum coverage of mixed aerobic and anaerobic pathogens. Two blood culture bottles (aerobic and anaerobic) were collected in the emergency department; the anaerobic bottle grew a gram-negative bacillus after approximately 24 hours, subsequently identified as *F. necrophorum*. Susceptibility testing showed the isolate was sensitive to clindamycin, cefmetazole, metronidazole, penicillin, ampicillin/sulbactam, piperacillin/tazobactam, meropenem, and doxycycline. Bone marrow biopsy was performed given the severity of thrombocytopenia to exclude a primary hematologic process; it demonstrated reactive myeloid hyperplasia, consistent with a consumptive peripheral etiology rather than primary marrow pathology. On day 5, once the organism and its pan-susceptible profile were confirmed, therapy was de-escalated from meropenem and doxycycline to piperacillin/tazobactam and metronidazole. Percutaneous drainage was deferred given the small lesion size and rapid clinical response. Platelets rose from 14 × 10^3^/μL on admission to 95 × 10^3^/μL by day 5 and normalized at 393 × 10^3^/μL by discharge. Inflammatory markers and bilirubin resolved in parallel. Follow-up ultrasound on day 7 confirmed complete hepatic lesion resolution, and the patient was discharged on day 12 on oral metronidazole 250 mg four times daily for 7 days and moxifloxacin 400 mg once daily for 14 days. Because contrast-enhanced abdominal and pelvic imaging had already excluded bowel or rectal wall thickening and an intra-abdominal or pelvic abscess, and the patient had no abdominal or anorectal symptoms, melena, or hematochezia, endoscopic evaluation was not pursued. At two-week infectious disease follow-up, there were no new events; a right lower lung lesion tracked on serial chest radiographs had subsided, and follow-up abdominal imaging confirmed a resolved liver abscess with stable hepatic vein thrombophlebitis and no new emboli. The patient had a full recovery with no complications or sequelae at this follow-up point.

## Discussion

This case represents a rare visceral variant of Lemierre's syndrome in which the intra-abdominal venous system served as the substrate for septic thrombophlebitis, mirroring the pathophysiology of classic jugular vein involvement. *F. necrophorum*, an obligate anaerobic gram-negative rod, elaborates potent endotoxins, hemolysins, and platelet-aggregating factors capable of inducing locoregional coagulation and metastatic embolic seeding [5]. The temporal and anatomic relationship between the hepatic abscess, the adjacent hepatic vein thrombophlebitis, and the bilateral peripheral wedge-shaped pulmonary nodules is most consistent with the liver-derived focus acting as the embolic source of the septic pulmonary emboli; however, this remains a clinical and radiologic inference rather than a directly proven mechanism, and an alternative unrecognized primary source cannot be entirely excluded.

Nomenclature and diagnostic reasoning merit explicit discussion, since the absence of pharyngitis in this patient could reasonably raise the question of whether the label "Lemierre's syndrome" is appropriate at all. We acknowledge that this patient never had sore throat, and we do not propose an oropharyngeal origin. Rather, we apply the term in its now widely used variant sense: a primary anaerobic focus, contiguous septic thrombophlebitis of the draining vein, and metastatic septic embolization arising from that infected thrombus. In this patient all three elements were present, with the hepatic vein substituting for the internal jugular vein. An alternative and entirely reasonable mechanistic sequence, and one we considered, is that anorectal mucosal inoculation during receptive anal intercourse produced a clinically silent proctocolitis, with portal venous translocation of Fusobacterium seeding the liver, formation of the hepatic abscess, secondary hepatic vein thrombophlebitis, and finally embolization through the inferior vena cava and right heart to the pulmonary arteries. We emphasize that this proposed pathway does not conflict with the diagnosis; it is precisely the mechanism that defines the visceral variant, in which the splanchnic venous system replaces the cervical venous system as the route of dissemination. Whether the portal of entry was anorectal, another gastrointestinal site, or occult, the syndromic construct and its clinical implications remain the same.

We nonetheless sought evidence for an anorectal or gastrointestinal portal of entry and did not find it. The patient denied anorectal pain, tenesmus, discharge, bleeding, diarrhea, and abdominal pain; perianal and digital rectal examination were unremarkable; and contrast-enhanced CT of the abdomen and pelvis showed no rectal or colonic wall thickening, pericolic stranding, perirectal collection, portal or mesenteric venous thrombus, or periportal gas. Sexually transmitted infection screening, including HIV and syphilis serology, was negative. A genitourinary source was likewise considered, with no imaging evidence of nephritis or prostatitis. Endoscopic and anoscopic evaluation were not performed, and this is a limitation: a subclinical proctitis cannot be formally excluded, and nucleic acid amplification testing of a rectal swab for *Neisseria gonorrhoeae* and *Chlamydia trachomatis* was not obtained. In a patient with comparable epidemiologic risk, we would now recommend rectal nucleic acid amplification testing and a low threshold for anoscopy, even in the absence of anorectal symptoms, since establishing the portal of entry has both prognostic and public health value. The definitive portal of entry in this case therefore remains unidentified, which is not unusual in the visceral variant of Lemierre's syndrome, where the primary focus is reported as occult in a substantial minority of cases.

The severe thrombocytopenia in this critically ill patient prompted evaluation for a hematologic process, and bone marrow biopsy was undertaken to exclude primary marrow pathology; in retrospect, the rapid and sustained platelet recovery that paralleled clinical improvement was itself strongly suggestive of a consumptive, sepsis-related etiology, and a period of watchful waiting with serial platelet counts may have obviated the need for biopsy in a similar future presentation. A single positive lupus anticoagulant (dRVVT normalized ratio 1.29; reference < 1.21) was identified during acute illness; infection-associated lupus anticoagulant is a recognized phenomenon, and endothelial disruption with transient antiphospholipid antibody generation has been described during acute Fusobacterium infection [6], but transience has not been demonstrated in this patient, since only one measurement is available. We therefore describe this as a suspected infection-associated lupus anticoagulant rather than a confirmed transient phenomenon. Repeat serologic testing at three months, intended to confirm resolution, had not yet been performed at the time of writing.

Hepatic vein thrombophlebitis was a key feature of this case, and the decision regarding anticoagulation warrants explicit discussion. Anticoagulation was not administered. This decision reflected the infectious etiology of the thrombophlebitis, the expectation that antimicrobial therapy would address the primary driver of thrombus formation, and the bleeding risk posed by a platelet count of 14 × 10^3^/μL on admission. Anticoagulation in septic thrombophlebitis, including classic Lemierre's syndrome, remains controversial and is not routinely recommended in the absence of thrombus propagation or clot in a critical location. On follow-up imaging, the hepatic vein thrombophlebitis remained stable without progression or new embolic events, and liver function normalized in parallel with antimicrobial therapy alone.

Empiric therapy was initiated with meropenem and doxycycline for broad-spectrum coverage while cultures were pending. Once blood cultures identified *F. necrophorum*, therapy was de-escalated on day 5 to piperacillin/tazobactam and metronidazole, a combination chosen on the basis of clinical judgement and in vitro susceptibility testing.

Conservative, antibiotic-only management was pursued for this small hepatic abscess, with percutaneous drainage deferred given the size of the lesion and the rapid clinical response. Limited evidence, drawn largely from case reports and small case series rather than controlled studies, suggests that antibiotic-only therapy can be effective for small pyogenic liver abscesses caused by Fusobacterium species when early clinical and radiographic improvement is observed, reserving drainage for larger collections or treatment failure [7, 8].

Most previously reported abdominal variants of Lemierre's syndrome involve septic thrombophlebitis of the portal or mesenteric venous system (pylephlebitis) rather than the hepatic vein itself; a recent systematic review identified 36 reported cases of the gastrointestinal variant of Lemierre's syndrome, and other literature reviews describe more than twenty cases of Fusobacterium-associated pylephlebitis [8]. True hepatic vein thrombophlebitis due to Fusobacterium is considerably rarer and has been described mainly in isolated reports [5]. The present case, with hepatic rather than portal or mesenteric vein thrombophlebitis, adds to this small body of literature and highlights that the visceral variant of Lemierre's syndrome extends beyond the portal venous system.

Clinicians evaluating young immunocompetent patients with polymetastatic septic presentations, severe cytopenias, and no oropharyngeal source should obtain cross-sectional chest and abdominal imaging and ensure that blood culture protocols include anaerobic identification technique and extended anaerobic incubation, since *F. necrophorum* grows slowly and may be missed by standard processing windows if anaerobic cultures are not specifically requested or incubated long enough; this consideration is one of the most actionable diagnostic points raised by this case. In patients with relevant sexual exposure history, anorectal mucosal disease should be actively sought as a candidate portal of entry, even when anorectal symptoms are absent.
